# Prevalence and factors associated with anaemia among adolescents in Sub-Saharan Africa and India

**DOI:** 10.1371/journal.pgph.0006963

**Published:** 2026-07-31

**Authors:** Adom Manu, Rutuja Patil, Isaac Boadu, Justice Moses Aheto, Sachin Shinde, Esther Abandoh-Sam, Elizabeth Awini, Emmanuel Anongeba Anaba, Emmanuel Darkwa, Kenneth Nartey, Diksha Singh, Yula Salifu, Amani Tinkasimile, Nega Assefa, Angela Chukwu, Justine Bukenya, Ourohire Millogo, Mary Mwanyika-Sando, John Williams, Till Bärnighausen, Wafaie Fawzi

**Affiliations:** 1 Department of Population, Family and Reproductive Health, University of Ghana School of Public Health , Accra, Ghana; 2 Vadu Rural Health Program, KEM Hospital Research Centre, Pune, India; 3 Harvard T. H. Chan School of Public Health, Boston, Massachusetts, United States of America; 4 Dodowa Health Research Centre, Ghana Health Service, Dodowa, Ghana; 5 Africa Academy for Public Health, Dar es Salaam, Tanzania; 6 College of Health and Medical Sciences, Haramaya University, Addis Ababa, Ethiopia; 7 Department of Statistics, University of Ibadan, Ibadan, Nigeria; 8 School of Public Health, Makerere University, Kampala, Uganda; 9 Centre de Recherche en Santé de Nouna, Nouna, Burkina Faso; 10 Heidelberg Institute of Global Health, Heidelberg University, Heidelberg, Germany; World Health Organization Regional Office for South-East Asia, INDIA

## Abstract

Anaemia remains a major global health concern among adolescents. While regional and national studies report varying prevalence, cross-continental evidence is limited. This study examines anaemia prevalence and its determinants among adolescents in Sub-Saharan Africa and India. We analyzed cross-sectional data from 9,820 adolescents (10–19 years) in Burkina Faso, Ethiopia, Ghana, India, Nigeria, Tanzania, and Uganda^.^ Hemoglobin levels were measured using a finger-prick capillary blood and a portable HemoCue device, with anaemia defined according to the World Health Organization’s age- and sex-specific cutoffs. Multivariable logistic regression was used to identify factors associated with anaemia. The sample comprised 51.5% females; 56.8% had ≥ 7 years of education, and 91.5% were literate. The mean age was 14.2 years (SD ± 2.7), with 52.8% aged 10–14 years. Overall, anaemia prevalence was 42.0% (95% CI: 40.9-43.1), including 3.9% with severe anaemia. Prevalence varied substantially by country; lowest in Ethiopia (4.9%) and highest in Ghana (73.6%). While the prevalence of anaemia across Sub-Saharan African countries was 43.9%, it was 31% in India. Overall, female adolescents had 15% higher odds of anaemia than males (adjusted odds ratio [aOR] 1.15; 95% CI: 1.04–1.28), while older adolescents 15–19 years had lower odds compared with younger adolescents 10–14 years (aOR 0.87; 95% CI: 0.69–0.94). Adolescents with normal (aOR 0.76; 95%CI: 0.64-0.90) and overweight (aOR 0.79; 95%CI: 0.63-0.97) body mass index (BMI) had lower odds of anaemia than their underweight counterparts. Consumption of eggs and legumes was protective, and residing in Ghana was associated with doubled odds of anaemia (aOR 2.21; 95%CI: 1.74–2.80). Anaemia remains highly prevalent among adolescents in Sub-Saharan Africa and India, particularly among females. Protective correlation of consuming eggs and legumes highlights the potential of targeted feeding interventions. The higher prevalence in younger adolescents underscore the need for age-specific strategies to combat adolescent anaemia. Additionally, underweight status is a risk factor for anaemia; thus, nutrition literacy intervention is imperative for improved nutritional status to reduce adolescent anaemia.

## Introduction

Anaemia is a global public health challenge and ranks among the leading causes of Disability Adjusted Life Years (DALYs) worldwide [1,2]. Approximately 1.92 billion people globally suffer from anaemia, with the greatest burden concentrated in low and middle-income countries (LMICS) [1], where systemic health inequities, nutritional deficiencies, and infectious diseases compound the problem. Historically, children under five years and women of reproductive age have borne the highest burden and have consequently been the primary focus of anaemia prevention and control programs [2,3]. However, this focus has inadvertently left a critical population of adolescents aged 10–19 years, largely overlooked despite their substantial and growing burden of anaemia [4,5].

Adolescence represents a critical developmental window characterized by rapid physical, cognitive, and psychosocial development, all of which create heightened nutritional demands [6,7]. During this period, individuals experience accelerated bone and muscle development, increased blood volume, and hormonal changes that substantially elevate requirements for iron, folate, and other micronutrients. For adolescent girls, the onset of menstruation further amplifies iron needs, making them particularly vulnerable to anaemia [6,8].

Beyond biological factors, adolescents face unique behavioral and environmental challenges that compromise their nutritional status. This stage of life is also marked by heightened psychosocial stressors, including academic pressures, changing social roles, gender norms, and increasing expectations around autonomy and responsibility. These psychosocial factors can shape food-related decision-making, health-seeking behaviours, and adherence to nutrition and supplementation programmes, particularly in low-resource settings [9,10]. As adolescents gain independence, dietary choices are increasingly influenced by peer norms, school environments, and householder power dynamics rather than caregiver control. As they gain autonomy over food choices, many are exposed to unhealthy school and community food environments that promote energy-dense, nutrient-poor foods while limiting access to diverse, micronutrient-rich diets [9–11]. Poor dietary diversity, combined with limited nutrition knowledge, exacerbates their vulnerability to micronutrient deficiencies. Additionally, many adolescents in LMICs lack access to adolescent-friendly health services that could provide nutritional counseling, iron supplementation, or early detection of anemia [6,12,13].

The consequences of anaemia during this life stage are profound and far-reaching. Anaemia impairs cognitive function, reduces physical work capacity, compromises academic performance, and increases susceptibility to infections and other morbidities [2,14,15]. Critically, nutritional deficits acquired during adolescence often persist into adulthood, perpetuating an intergenerational cycle of poor health, particularly when adolescent girls transition into pregnancy and motherhood with anaemia [6].

Sub-Saharan Africa (SSA) and South Asia represent two regions where the burden of adolescent anaemia is exceptionally high and where the intersection of poverty, food insecurity, infectious diseases, and weak health systems creates a perfect storm for persistent micronutrient deficiencies. According to the Global Burden of Disease Study, anaemia prevalence among adolescents in SSA and South Asia remains the highest globally, with rates exceeding 40% in many settings [1]. In SSA, countries such as Burkina Faso, Ghana, Mali, Niger, Nigeria, Tanzania and Uganda carry a disproportionate share of this burden [1,16,17], while India continues to be the predominant contributor in South Asia, especially among adolescent girls [18–21]. Even with national programs such as the Anaemia Mukt Bharat (Anaemia-Free India) programme in India [22], anaemia rates remain persistently high.

National data from the Comprehensive National Nutrition Survey (2016–2018) estimated that about 72 million adolescents suffered from anaemia in India [3]. A meta-analysis of over 150,000 adolescent girls (10–19 years) across India reported a higher pooled prevalence of 65.7% of anaemia, with significant variations across states and between rural and urban settings [18]. Similarly, in SSA, a pooled analysis of Demographic and Health Surveys across 31 countries revealed that 42% of women aged 15–24 had anaemia [23], while a systematic review focusing specifically on adolescent girls in East and West Africa documented a pooled prevalence of 32.9% [24]. These regional statistics underscore the magnitude of the problem and the urgent need for context-specific research and interventions that address the unique needs of adolescents that drive anaemia in these settings.

Despite the mounting evidence of the high burden of anaemia among adolescents in SSA and India, significant knowledge gaps persist, limiting the design and implementation of effective interventions [3]. First, national health surveys and surveillance systems often underrepresent adolescents as a distinct demographic group, instead aggregating them with children and adults, obscuring age- and sex-specific patterns of anaemia prevalence and risk. Second, there is a limited understanding of the complex and context-specific determinants of adolescent anaemia beyond well-established factors of iron deficiency and dietary inadequacy. Emerging challenges such as climate change, which affects food production, crop diversity, and nutrient density, along with urbanization that reshapes food environments and nutrition transition toward processed and ultra-processed foods, remain poorly studied.

Third, robust, high-quality data on adolescent anaemia remains scarce in LMICs, with many studies relying on small, non-representative samples. Fewer studies have adopted cross-continental comparative approaches that would enable researchers to identify common risk and protective factors across diverse settings. Finally, existing anaemia reduction efforts in SSA and India are often fragmented, vertically structured, and focused primarily on women and children, with adolescent-specific interventions remaining limited in scope and coverage [3,25–27].

In this context, this study examines the prevalence of anaemia and its determinants among adolescents (aged 10–19 years) across six SSA countries (Burkina Faso, Ethiopia, Ghana, Nigeria, Tanzania, and Uganda) and India. By pooling data from multiple countries and employing rigorous analytical methods, this study provides a comprehensive, cross-sectional perspective on adolescent anaemia that is largely absent from the literature. The findings aim to inform policymakers, public health professionals, and researchers about the scale and determinants of adolescent anaemia in high-burden settings and to guide the development of evidence-based, adolescent-specific nutrition interventions.

## Methods

### Ethics statement

The study was approved by the Institutional Review Committees or Boards across all the partner institutions as follows: Burkina Faso – Ministry of Health (Deliberation No. 2021-10-229); Ethiopia – Institutional Health Research Ethics Review Committee, Haramaya University College of Health and Medical Science (IHRERC/142/2021); Ghana – Ghana Health Service Ethics Review Committee (GHS-ERC: 018/10/21); India – KEM Hospital Research Centre Ethics Committee KEMHRC/RVM/EC/1482); Nigeria – Social Science and Humanities Research Ethics Committee, University of Ibadan (UI/SSHRC/2021/058); Tanzaina - the National Institute for Medical Research (NIMR/HQR.8a/Vol.IX/3974); Uganda – Makerere University School of Public Health (SPH-2021–156) and the Uganda National Council for Science and Technology (HS1906ES); and in the United States of America - Institutional Review Board of the Harvard T. H. Chan School of Public Health, Boston (IRB 21–0696). Additionally, written informed consent was obtained from adolescents aged 18–19 years, while parental consent and adolescent assent were obtained for participants aged 10–17 years. Participants were assured of privacy and confidentiality.

### Inclusivity in global research

Additional information regarding the ethical, cultural, and scientific considerations specific to inclusivity in global research is included in the Supporting Information (S1 Checklist).

### Study design and settings

This cross-sectional study analyzed data from the Africa Research, Implementation Science and Education (ARISE) Network longitudinal survey conducted between November 2021 and January 2024 [28]. The ARISE Network, established in 2014, comprises 22 member institutions from 10 SSA countries with collaborating partners in Europe, Asia, and North America [28,29]. Baseline data were collected from adolescents at eight sites in seven countries: Burkina Faso, Ethiopia, Ghana, India, Nigeria, Tanzania (Dar es Salaam and Tanga), and Uganda. Six sites utilized Health and Demographic Surveillance System (HDSS) platforms as sampling frames, while two sites (Nigeria and Tanga, Tanzania) conducted school-based surveys.

### Study population and sampling

The study enrolled 9,820 adolescents aged 10–19 years, approximately 1,200 participants per site. Participants were selected using stratified random sampling based on sex (male/female) and age category (10–14 years and 15–19 years) to ensure balanced representation across demographic subgroups. Detailed sampling procedures have been published previously [28].

### Data collection

Trained field workers administered a standardized ARISE Network adolescent health and well-being survey questionnaire covering socio-demographic characteristics, physical health, nutrition, mental health, substance use, sexual and reproductive health, and health services utilization. Questionnaire development and validation processes have been described elsewhere [28].

Hemoglobin (Hb) levels were measured using finger-prick capillary blood samples, analyzed with a portable HemoCue analyzer (HemoCue AB, Angelholm, Sweden). Across all study sites, medical laboratory scientists trained field workers in standardized finger-prick capillary blood sample collection, operation of the HemoCue analyzer, infection prevention and control, and quality assurance measures to ensure accurate and reliable haemoglobin tests. A finger-prick capillary blood sample was collected using a sterile, single-use lancet after cleaning and air-drying the puncture site. Thereafter, a free-flowing drop of blood was placed into a disposable microcuvette, which was immediately inserted into the HemoCue analyzer to determine the hemoglobin concentration in approximately one minute. The HemoCue analyzer was factory-calibrated, and its performance was verified daily using the manufacturer’s control cuvette for accuracy.

Anthropometric measurements (weight and height) were obtained using calibrated digital scales and stadiometers, respectively. Across all sites, data collection took place between November 2021 and July 2022. Specifically, recruitment of participants occurred from 07/03/2022 to 05/04/2022 in Burkina Faso; from 10/11/2021 to 20/12/2021 in Ethiopia; from 07/03/2022 to 29/03/2022 in Ghana; from 18/01/2022 to 21/04/2022 in India; from 11/05/2022 to 21/07/2022 in Nigeria; from 18/06/2022 to 30/07/2022 in Tanzania; and from 06/02/2022 to 23/02/2022 in Uganda.

### Outcome variable

#### Anaemia status.

The primary outcome was anaemia, defined according to World Health Organization (WHO) age- and sex-specific hemoglobin (Hb) thresholds [30]. For children aged 10–11 years, anaemia was defined as Hb < 11.5 g/dL, while for girls aged 12–19 years and boys aged 12–14 years, the threshold was < 12.0 g/dL. Boys aged 15–19 years were considered anaemic at Hb < 13.0 g/dL. Anaemia severity was classified as mild, moderate, or severe based on WHO criteria: mild anaemia was defined as Hb 11.0–11.4 g/dL for 10–11 year-olds, 11.0–11.9 g/dL for girls 12–19 years and boys 12–14 years, and 11.0–12.9 g/dL for boys 15–19 years; moderate anaemia as Hb 8.0–10.9 g/dL for all groups; and severe anaemia as Hb < 8.0 g/dL across all age and sex categories [30]. A binary variable (anaemic/non-anaemic) was used for primary analyses.

### Predictors

#### Global Diet Quality Score (GDQS).

Diet quality was assessed using the validated GDQS, a food-based metric comprising 25 food groups: 16 healthy groups, 7 unhealthy groups, and 2 groups (red meat and high-fat dairy) that are unhealthy in excessive amounts [31]. Participants reported consumption frequency of each food group over the past week, with responses coded as “0–1 time per week” = 0 points, “2–3 times per week” = 1 point, and “4 or more times per week” = 2 points. For high-fat dairy, an additional category (≥ 3 times/week or >4times/week) was coded as 3 points. Scoring is assigned higher points for greater consumption of healthy foods and lower points for unhealthy foods. The composite GDQS score ranged from 0–49 and was categorized as: high risk (< 15), medium risk (15–23) and low risk (>23) for poor diet quality.

#### Body Mass Index (BMI).

BMI was calculated as weight (kg)/ height (m^2^). To assess nutritional status, BMI-for-age Z-scores (BAZ) were computed using the WHO 2007 growth reference standards for children and adolescents aged 5–19 years, using the WHO AnthroPlus software. Adolescents were classified as underweight (BAZ <−2 standard deviations (SD)), normal weight (−2 SD to +1 SD), overweight (+1 to +2 SD), or obese (>/ + 2 SD) [32].

#### Other predictors.

Sociodemographic variables included age, sex, country of residence, literacy status, year of education, subjective social status, health insurance coverage, and alcohol consumption. For female participants, age at menarche was also recorded. Individual food group consumption frequencies from the GDQS (e.g., eggs, legumes, fish, dark green leafy vegetables) were examined as separate predictors.

### Statistical analysis

Data from all eight sites were pooled, cleaned and analyzed using Stata 17.0 (StataCorp, College Station, TX, USA). Descriptive statistics summarized participant characteristics, with categorical variables presented as frequencies and proportions, and continuous variables as means and standard deviations. Chi-square tests assessed bivariate associations between anaemia and independent variables. Multivariable logistic regression models were developed using a stepwise approach to identify factors associated with anaemia. Model 1 examined the association between GDQS and anaemia. Model 2 added sociodemographic variables (sex, age, BMI, health insurance, subjective social status, and country) while retaining GDQS. Model 3 (final model) replaced the composite GDQS with individual food groups alongside sociodemographic predictors. The final model was selected based on predictive performance and clinical interpretability. Multicollinearity was assessed using variance inflation factors (VIF). All VIF values were within acceptable thresholds, indicating no evidence of significant multicollinearity in the fitted model. Adjusted odds ratios (aORs and 95% confidence intervals (CI) quantified associations, with statistical significance set at p < 0.05.

## Results

### Socio-demographic characteristics of the study participants

The study included 9,820 adolescents with a mean age of 14.2 years (SD ± 2.7). Females comprised 51.5% of the sample, and 52.8% were aged 10–14 years. Most participants were literate (91.5%), with 40.6% having completed 1–6 years of education and 31.6% having completed 7–9 years. Among female participants (n = 5,059), the mean age of menarche was 13.0 years (SD ± 1.3). Most participants (73.0%) self-identified as middle socioeconomic status. Table 1 shows participants’ sociodemographic characteristics.

**Table 1:** Socio-demographic characteristics of participants in SSA and India.

Characteristic	Frequency (%)
**Age (Years)**	Mean (SD): 14.2 (2.7)
10–14 years	5173 (52.8)
15–17 years	3192 (32.6)
18–19 years	1440 (14.7)
**N**	9805
**Sex**	
Male	4756 (48.5)
Female	5059 (51.5)
**N**	9815
**Age at Menarche (Years)**	Mean (SD): 13.2 (1.3)
8–10	63 (2.0)
11–12	877 (27.9)
13–14	1766 (56.2)
15–19	434 (13.8)
**N**	3140
**Years of Education**	
0	210 (2.5)
1–6	3376 (40.6)
7–9	2628 (31.6)
10–12	1958 (23.5)
13–16	144 (1.7)
**N**	8316
**Country**	
Burkina Faso	1202 (12.2)
Dar es Salaam, Tanzania	1201 (12.2)
Ethiopia	1200 (12.2)
Ghana	1245 (12.7)
India	1246 (12.7)
Nigeria	1264 (12.9)
Tanga, Tanzania	1262 (12.9)
Uganda	1200 (12.2)
**Total N**	9820
**Subjective Social Status**	
Low	1965 (20.0)
Middle	7159 (73.0)
High	689 (7.0)
**N**	9813
**Able to Read and Write**	
No	492 (5.7)
Can read	112 (1.3)
Can write	124 (1.4)
Can read and write	7868 (91.5)
**N**	8596
**Alcohol intake (past 30 days)**	
0–3 days	1001 (76.8)
4–6 days	187 (14.3)
7 or more	118 (9.0)
**N**	1306
**BMI-for-age (WHO)**	
Normal	7644 (79.4)
Thinness	944 (9.8)
Overweight/Obese	1039 (10.8)
**N**	9627
**Legumes ≥4 times/week**	
No	7749 (98.0)
Yes	159 (2.0)
**N**	7908
**Other vegetables ≥4 times/week**	
No	7880 (99.9)
Yes	8 (0.1)
**N**	7888
**Nuts and seeds ≥4 times/week**	
No	7693 (97.4)
Yes	203 (2.6)
**N**	7896
**Unprocessed red meat ≥4 times/week**	
No	7874 (99.9)
Yes	8 (0.1)
**N**	7882
**Processed red meat ≥4 times/week**	
No	7878 (99.9)
Yes	3 (0.1)
**N**	7881
**Poultry/game meat ≥4 times/week**	
No	7878 (99.9)
Yes	3 (0.1)
**N**	7881
**Fish/shellfish ≥4 times/week**	
No	7880 (99.9)
Yes	3 (0.1)
**N**	7883
**Eggs ≥4 times/week**	
No	7866 (99.6)
Yes	30 (0.4)
**N**	7896
**Low-fat dairy ≥4 times/week**	
No	7860 (99.5)
Yes	41 (0.5)
**N**	7901
**Health Insurance**	
No	7676 (78.6)
Yes	2083 (21.4)
**N**	9759

Note: All sample sizes are below the total study population of 9,820 participants. The “No” category for dietary variables combines consumption frequencies of 0–3 times per week.

### Anaemia prevalence

**Fig 1:**
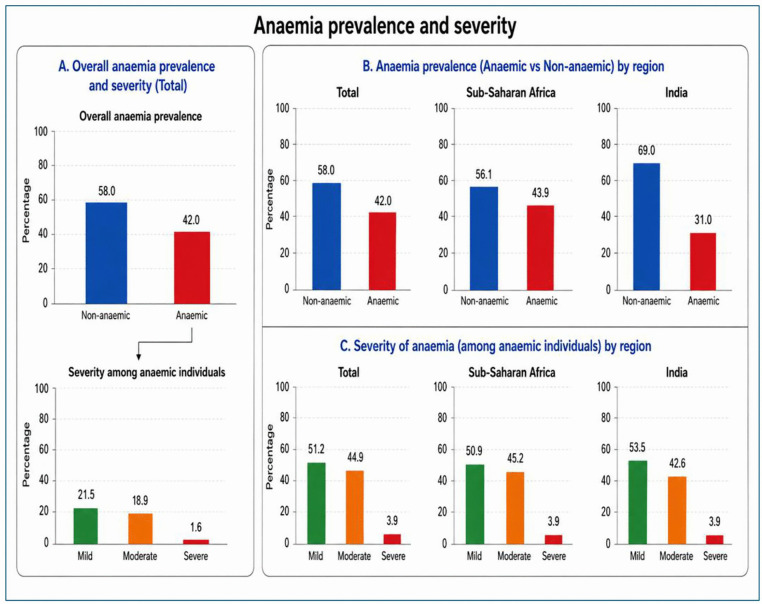
Anaemia prevalence and severity among participants by region. **A.** Overall anaemia prevalence and severity, **B.** Anaemia prevalence (Anaemic vs Non-Anaemic) by region, **C.** Severity of anaemia (among anaemic individuals) by region.

The overall prevalence of anaemia was 42% (95% CI:40.9-43.1), with 21.5% classified as mild, 18.9% as moderate, and 1.6% as severely anemic among 9820 adolescents. While the prevalence of anaemia across Sub-Saharan African countries was 43.9%, it was 31% in India. Overall, among those with anaemia, 3.9% had severe anaemia. Fig 1 (Panels A, B and C) provides details of the distribution of anaemia prevalence and severity in SSA and India is shown in Fig 1. Prevalence varied substantially by country, ranging from 4.9% in Ethiopia to 73.6% in Ghana (Table 2). Anaemia was more prevalent among females (42.6% vs 40.9% in males), adolescents aged 15–17 years (44.5%), those with no formal education (56.0%), and those classified as underweight based on BMI-for-age (44.1%).

**Table 2:** Association of participant characteristics with anaemia.

Variable/ Category	Non-Anaemic (%)	Anaemic (%)	*p*-value
**Age (years)**			<0.001
10–14	2939 (57.5)	2177 (42.5)	
15–17	1725 (55.5)	1386 (44.5)	
18–19	945 (62.3)	460 (37.7)	
**Total**	5609 (58.2)	4023 (41.8)	
**Sex**			0.09
Male	2751 (59.1)	1906 (40.9)	
Female	2860 (57.4)	2125 (42.6)	
**Total**	5611 (58.2)	4031 (41.8)	
**Years of Education**			<0.001
0	92 (44.0)	117 (56.0)	
1–6	1960 (58.4)	1395 (41.6)	
7–9	1414 (54.8)	1165 (45.2)	
10–12	1214 (63.0)	713 (37.0)	
13–16	89 (61.8)	55 (38.2)	
**Total**	4769 (58.1)	3445 (41.9)	
**Country**			<0.001
Burkina Faso	514 (42.8)	688 (57.2)	
Dar Tanzania	551 (48.2)	593 (51.8)	
Ethiopia	1136 (95.1)	59 (4.9)	
Ghana	327 (26.4)	912 (73.6)	
India	859 (69.1)	385 (30.9)	
Nigeria	481 (39.2)	746 (60.8)	
Tanga Tanzania	734 (61.3)	464 (38.7)	
Uganda	1013 (84.5)	186 (15.5)	
**Total**	5615 (58.2)	4033 (41.8)	
**Subjective Social Status**			0.04
Low	1127 (58.6)	797 (41.4)	
Middle	4116 (58.5)	2916 (41.5)	
High	367 (53.7)	317 (46.3)	
**Total**	5610 (58.2)	4030 (41.8)	
**Days of alcohol intake**			<0.001
0–3 days	447 (44.7)	554 (55.3)	
4–6 days	105 (56.2)	82 (43.8)	
7 or more	68 (63.6)	39 (36.5)	
**Total**	620 (47.9)	675 (52.1)	
**BMI-for-age**			0.27
Normal	4469 (58.6)	3154 (41.4)	
Thinness	527 (55.9)	416 (44.1)	
Overweight/Obese	602 (57.9)	437 (42.1)	
**Total**	5598 (58.3)	4007 (41.7)	
**Able to read and write**			<0.001
No	225 (45.7)	267 (54.3)	
Can read	58 (51.8)	54 (48.2)	
Can write	59 (47.6)	65 (52.4)	
Can read & write	4661 (59.2)	3207 (40.8)	
**Total**	5003 (58.2)	3593 (41.8)	
**Legumes ≥4 times/week**			0.15
No	5452 (58.2)	3914 (41.8)	
Yes	102 (52.3)	93 (47.7)	
**Total**	5554 (58.2)	4007 (41.8)	
**Other vegetables ≥4 times/week**			0.78
No	5548 (58.2)	3989 (41.8)	
Yes	5 (55.6)	4 (44.4)	
**Total**	5553 (58.2)	3993 (41.8)	
**Nuts & seeds ≥4 times/week**			<0.001
No	5412 (58.3)	3876 (41.7)	
Yes	118 (53.2)	104 (46.8)	
**Total**	5530 (58.2)	3980 (41.8)	
**Unprocessed red meat ≥4 times/week**			0.90
No	5548 (58.2)	3989 (41.8)	
Yes	5 (62.5)	3 (37.5)	
**Total**	5553 (58.2)	3992 (41.8)	
**Processed red meat ≥4 times/week**			0.38
No	5545 (58.2)	3986 (41.8)	
Yes	3 (100.0)	0 (0.0)	
**Total**	5548 (58.2)	3986 (41.8)	
**Poultry/game ≥4 times/week**			0.25
No	5545 (58.2)	3986 (41.8)	
Yes	3 (100.0)	0 (0.0)	
**Total**	5548 (58.2)	3986 (41.8)	
**Fish/shellfish ≥4 times/week**			0.55
No	5546 (58.2)	3987 (41.8)	
Yes	2 (66.7)	1 (33.3)	
**Total**	5548 (58.2)	3988 (41.8)	
**Eggs ≥4 times/week**			0.01
No	5540 (58.2)	3985 (41.8)	
Yes	18 (60.0)	12 (40.0)	
**Total**	5558 (58.2)	3997 (41.8)	
**Low-fat milk/dairy ≥4 times/week**			<0.001
No	5532 (58.2)	3979 (41.8)	
Yes	26 (54.2)	22 (45.8)	
**Total**	5558 (58.2)	4001 (41.8)	
**Health Insurance**			<0.001
No	4322 (56.3)	3354 (43.7)	
Yes	1297 (62.3)	786 (37.7)	
**Total**	5619 (57.6)	4140 (42.4)	

Note: Percentages are row percentages. The totals in each section may not sum to the overall study N (9,821 due to missing data for specific variables. The analysis uses all available data per variable.

### Bivariate associations with anaemia

Chi-square tests revealed significant associations (*p* < 0.05) between anaemia and country of residence, age, age at menarche, years of formal education, and subjective social status (Table 2). Nutritional status (BMI-for-age category) and several food groups also showed significant bivariate association with anaemia.

### Multivariable predictors of anaemia

**Fig 2:**
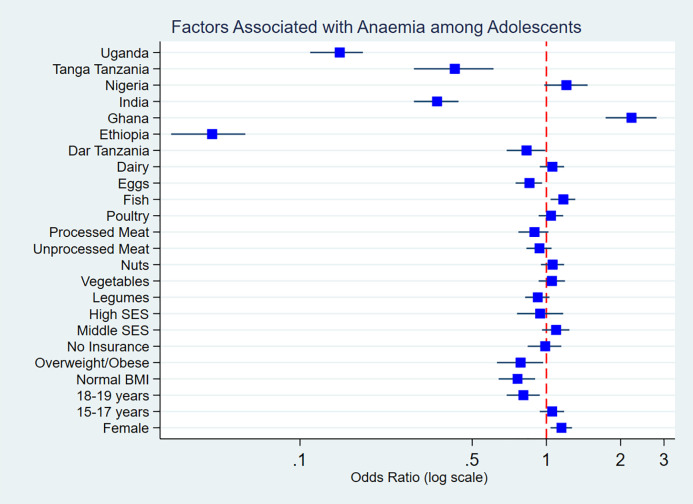
Plot of odds ratios and their 95% confidence intervals for the multivariable logistic regression model.

Three sequential logistic regression models were constructed to identify independent predictors of anaemia. Model 1 examined the GDQS alone and found that adolescents at low risk for poor diet quality had 31% higher odds of anaemia compared to those with normal diet quality (OR=1.31, 95% CI: 1.21–1.42, p < 0.001). In Model 2, which included GDQS alongside sociodemographic variables (sex, age, BMI, health insurance, subjective social status, and country), GDQS was no longer statistically significant, while sex, age, BMI, and country emerged as significant predictors. Model 3 (final model) replaced the composite GDQS with individual food groups and found significant associations between a few food groups and anaemia status, alongside sociodemographic predictors. Results from the final model are presented below in **Fig 2** and **Table 3**. **Results from the multivariable binary logistic regression.**

**Table 3:** Results from the multivariable binary logistic regression.

Variables	Odds ratio	St. Err.	t-value	p-value	[95%CI]
**Sex**					
**Male**	1				
Female	1.152	0.058	2.79	0.005	1.04–1.27
**Age**					
10-14	1				
15–17yrs	1.056	0.067	0.96	0.335	0.94–1.18
18–19yrs	0.807	0.062	−2.74	0.006	0.69–0.94
**BMIZ**					
Thinness	1				
Normal	0.764	0.064	−3.20	0.001	0.64–0.90
Overweight/obese	0.786	0.087	−2.15	0.032	0.63–0.97
**Have Insurance**					
Yes	1				
No	0.989	0.079	−0.13	0.899	0.84–1.15
**Social status**					
Low	1				
Middle	1.095	0.072	1.38	0.168	0.96–1.24
High	0.944	0.103	−0.52	0.605	0.76–1.17
**Legumes**					
No	1				
Yes	0.923	0.053	−1.37	0.070	0.82–1.03
**Other vegetables**					
No	1				
Yes	1.053	0.065	0.84	0.403	0.93–1.19
**Nuts and seeds (incl. nut butter)**					
No	1				
Yes	1.06	0.059	1.90	0.277	0.95–1.18
**Unprocessed red meat**					
No	1				
Yes	0.938	0.054	−1.10	0.272	0.83–1.05
**Processed red meat**					
No	1				
Yes	0.895	0.063	−1.55	0.122	0.77–1.02
**Poultry and any game meat**					
No	1				
Yes	1.045	0.062	0.76	0.449	0.93–1.17
**Fish and shellfish**					
No	1				
Yes	1.172	0.069	2.70	0.007	1.04–1.31
**Eggs**					
No	1				
Yes	0.854	0.053	−2.52	0.012	0.75–0.96
**Low-fat milk and dairy products**					
No	1				
Yes	1.058	0.062	0.95	0.342	0.94–1.18
**Country**					
Burkina Faso	1				
Dar Tanzania	0.831	0.077	−1.98	0.048	0.69–0.99
Ethiopia	0.044	0.007	−19.48	0.000	0.03–0.06
Ghana	2.216	0.267	6.59	0.000	1.74–2.80
India	0.360	0.036	−9.95	0.000	0.29–0.44
Nigeria	1.206	0.122	1.85	0.064	0.98–1.47
Tanga Tanzania	0.425	0.080	−4.53	0.000	0.29–0.61
Uganda	0.145	0.015	−17.56	0.000	0.11–0.18
Mean dependent var	0.419	SD dependent var			0.493
Pseudo r-squared	0.190	Number of obs			8482
Chi-square	2197.93	Prob > chi2			0.000
Akaike crit. (AIC)	9384.443	Bayesian crit. (BIC)			9560.585

**NB**: Food groups used are those found to be associated with anaemia, **yes** -means participants consumed 2–3 times/ week or more than 4 times/week, but less than 3times/day. No means 0–1 times/week; Low SSS (1–3), middle (4–7), high SSS (8–10).

#### Sociodemographic predictors.

Female adolescents had 15% higher odds of anaemia compared to males (aOR=1.15, 95% CI: 1.04–1.27, p < 0.001). Compared to adolescents aged 10–14 years, those aged 18–19 years had 20% lower odds of anaemia (aOR=0.80, 95% CI:0.69–0.94, p < 0.001). Adolescents with normal BMI had 30% lower odds of anaemia compared to those classified as underweight (aOR=0.70, 95% CI: 0.64–0.90, p < 0.001).

#### Dietary factors.

Consumption of eggs (≥ times per week) was associated with 16% lower odds of anaemia (aOR=0.84, 95% CI: 0.75–0.96, p < 0.001). Legume consumption showed a protective trend but did not reach statistical significance in the final model.

#### Geographic variation.

Compared to Burkina Faso (reference), adolescents in Ghana had more than double the odds of anaemia (aOR=2.21, CI: 1.74–2.80, p < 0.001), while those in Nigeria had 20% higher odds (aOR=1.20, 95% CI: 0.98–1.47, p = 0.08). Ethiopia showed significantly lower odds of anaemia compared to the reference country (Table 3).

#### Model performance.

**Fig 3:**
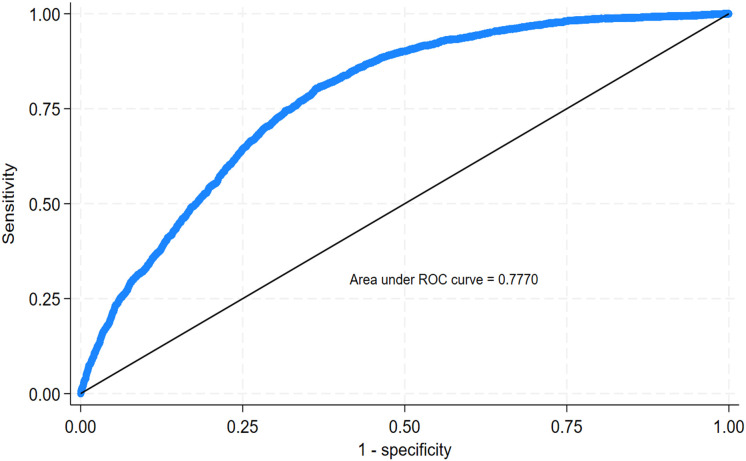
Area under ROC curve showing accuracy of logistic regression in predicting anaemia among study participants.

The final model demonstrated good discriminatory ability with an area under the receiver operating characteristic curve (AUROC) of 0.78 (95% CI: 0.76–0.80), indicating 78% correct classification of anaemia status (Fig 3). This suggests acceptable predictive performance for identifying adolescents at risk of anaemia based on the included sociodemographic, nutritional, and dietary variables.

#### Base outcomes.

Sex: Male | Age: 10–14 years | BMIZ: Thinness | Have Insurance: Yes | Social status: Low | Legumes: No | Other vegetables: No | Nuts and seeds: No | Unprocessed red meat: No | Processed red meat: No | Poultry and any game meat: No | Fish and shellfish: No | Eggs: No | Low fat milk and dairy products: No | Country: Burkina Faso.

**Accuracy of the multivariable logistic regression in predicting anaemia among study participants using the Area Under the Receiver Operating Characteristic (ROC) curve (** **Fig 3** **).**

## Discussion

This cross-continental study examined the prevalence of anaemia and associated factors among 9,821 adolescents aged 10–19 years across seven countries, six in SSA and India. Three principal findings emerged. First, the overall anaemia prevalence was 41.8%, with substantial geographic variation ranging from 4.9% in Ethiopia to 73.6% in Ghana [15,33]. Second, female sex, younger age (10–14 years), and underweight nutritional status were independent risk factors for anaemia [23,34]. Third, egg consumption (≥2 times per week) demonstrated a protective association with anaemia [31]. These findings provide critical evidence on the burden and determinants of adolescent anaemia in high-prevalence settings and underscore the need for targeted, context-specific interventions [21,28].

The striking geographic heterogeneity in anaemia prevalence, with West African countries (Ghana 73.6%, Nigeria 60.8%, Burkina Faso 57.2%) bearing the highest burden compared to East Africa (Ethiopia 4.9%) and India, aligns with previous research demonstrating regional disparities in adolescent anaemia across Sub-Saharan Africa [16,24]. Also, a multi-country study among in-school adolescents reported similarly low prevalence in Ethiopia (10.8%) but high prevalence in Tanzania (58.3%) [33]. These findings should be interpreted in light of site-specific ecological and surveillance contexts, including differences in sampling platforms and environmental factors such as altitude. These disparities likely reflect complex interactions among multiple contextual factors. In West Africa, the convergence of high malaria transmission, endemic helminth infections, chronic food insecurity, limited dietary diversity, and inadequate access to iron supplementation programs creates a syndemic that drives persistently high anaemia rates [14,35].

Additionally, it is noteworthy that the markedly higher prevalence of anaemia observed in Ghana, Nigeria and Burkina Faso may partly be explained by the high burden of haemoglobinopathies and haemolytic anaemias, particularly sickle cell disease (SCD), which are highly prevalent in West Africa [36,37]. Indeed, West Africa is recognised as a global hotspot for SCD, with a substantial proportion of the population carrying the sickle cell trait [38]. The chronic haemolysis and recurrent complications associated with these disorders increase the risk of anaemia and may contribute significantly to the elevated prevalence estimates observed in these countries [39,40]. This underlying disease burden may therefore help explain some of the regional heterogeneity in anaemia prevalence observed across the present study.

Moreover, the contribution of haemoglobinopathies to anaemia prevalence may interact with other contextual factors common in West Africa, including malaria transmission, nutritional deficiencies, recurrent infections and limited access to healthcare, thereby exacerbating anaemia risk. In effect, variations in the prevalence of these conditions across countries may partly account for the differences observed between West Africa, East Africa and India.

Conversely, Ethiopia’s relatively low prevalence may be attributed to national nutrition programs, widespread deworming initiatives, lower malaria endemicity in highland areas where much of the population resides, and potentially better dietary practices. The moderate prevalence in India, despite extensive national programming [22], highlights persistent challenges in program implementation, adherence, and reach to vulnerable adolescent populations [3,20]. These findings emphasize the need for context-adapted interventions that address region-specific drivers of anaemia rather than one-size-fits-all approaches [16].

Female adolescents demonstrated 15% higher odds of anaemia compared to males, a finding consistent with the broader literature on adolescent anaemia [23,41]. This sex disparity is biologically plausible and well-documented: menstruation imposes substantial iron losses, which, combined with inadequate dietary iron intake and limited iron supplementation coverage, render adolescent girls particularly vulnerable [4,6,42]. Studies from Ethiopia, for instance, have shown that menstruating adolescents have approximately twice the odds of anaemia compared to non-menstruating peers [43]. While some studies from Tanzania, Sudan, and Ethiopia have reported higher anaemia prevalence in boys, potentially related to growth spurts and higher iron requirements [33], the preponderance of evidence supports greater vulnerability among girls. This finding reinforces the imperative for sex-specific anaemia prevention strategies that address the unique physiological needs of adolescent girls, including menstrual health education and iron-folic acid supplementation programs tailored to this population [44].

Contrary to expectations, older adolescents (18–19 years) had 20% lower odds of anaemia compared to early adolescents (10–14 years). This counterintuitive finding, given that menstruating older girls would be expected to have higher anaemia risk, suggests potential age-related differences in exposure to interventions and health-promoting behaviours [45]. Several explanations merit consideration. First, existing iron supplementation programs in the region, such as Ghana’s Girls’ Iron-Folate Tablet Supplementation (GIFTS) program, predominantly target secondary school students who are typically older adolescents, leaving primary school-aged children underserved [44]. Second, older adolescents may have greater nutrition literacy and access to health information through school-based health education programs, enabling better dietary choices. Third, older adolescents may have completed their peak growth velocity period and thus have somewhat reduced micronutrient demands compared with younger adolescents undergoing rapid pubertal development [6]. This finding illuminates a critical gap in current anaemia prevention programming: the systematic exclusion of early adolescents (10–14 years), who represent a vulnerable yet overlooked population [28].

Underweight adolescents (thin BMI-for-age) had significantly higher odds of anaemia compared to those with normal nutritional status, a finding consistent with previous research from Southern Ethiopia, where thin adolescent girls had higher odds of anaemia [46,47]. This association likely reflects shared underlying causes: chronic food insecurity, inadequate dietary diversity, and insufficient intake of both macro- and micronutrients [10,48]. Adolescents experiencing undernutrition are likely deficient not only in iron but also in protein, folate, vitamin B12, vitamin A, and other nutrients essential for erythropoiesis [14]. Moreover, undernutrition may impair immune function and increase susceptibility to infections, thereby exacerbating anaemia through inflammatory pathways [34]. However, the literature on the BMI-anaemia relationship shows some inconsistency, with certain studies reporting no association or even inverse relationships. This heterogeneity may reflect differences in the etiology of anaemia across contexts (nutritional vs infectious) [14]. Regardless, the strong association observed in this study underscores the need for integrated nutrition interventions that address both macronutrient and micronutrient deficiencies simultaneously, rather than vertical iron supplementation programs in isolation [21].

Egg consumption emerged as a protective factor against anaemia, with adolescents consuming eggs regularly showing 16% lower odds of anaemia. This finding is biologically plausible given that eggs are nutrient-dense foods providing high-quality protein, bioavailable iron, folate, vitamin B12, and vitamin A: all critical for red blood cell production and iron metabolism [31]. Previous research has similarly demonstrated positive associations between egg consumption and hemoglobin levels among children and adolescents. However, the literature shows inconsistency, with some studies finding no association, suggesting that the relationship between egg consumption and anaemia may be modified by overall dietary patterns and baseline nutritional status [10].

Unexpectedly, our study found fish consumption to be associated with increased odds of anaemia, a finding that contradicts nutritional expectations given fish’s iron and protein content. Similar counterintuitive findings have been reported elsewhere [49]. These paradoxical results may reflect several methodological limitations and contextual factors. First, our dietary assessment relied on food frequency recall without portion size estimation, which may introduce measurement error [50]. Second, residual confounding is possible if fish consumption serves as a marker for other dietary or socioeconomic factors not fully captured in our models. Third, fish consumption patterns may vary by species and preparation methods, with some fish types potentially inhibiting iron absorption due to calcium content. These inconsistent findings underscore the complexity of diet-anaemia relationships and the need for caution in making dietary recommendations based on observational data alone [31,48].

These findings have several important implications for policy and programming. First, the exceptionally high anaemia prevalence in Ghana, Nigeria, Burkina Faso, and Tanzania demands urgent implementation of comprehensive, multi-sectoral anaemia control strategies that integrate nutrition-specific interventions with nutrition-sensitive approaches addressing underlying determinants [12,21]. Second, regular school-based anaemia screening programs should be established in high-burden countries to enable early detection and monitoring of trends over time [28]. Third, existing school-based iron supplementation programs must be expanded to reach younger adolescents in primary schools, who currently fall outside the coverage of most programs despite their evident vulnerability [45]. Fourth, given the pronounced sex differences in anaemia risk, targeted interventions for adolescent girls, including menstrual health education and improved access to iron-folic acid supplements, warrant prioritization [43,44]. Fifth, integrated nutrition programs addressing both undernutrition and micronutrient deficiencies simultaneously should be developed and evaluated [34]. Finally, intensified nutrition education emphasizing consumption of iron-rich foods should be incorporated into school curricula and community-based adolescent health programs [10,31].

This study has several notable strengths. The large, geographically diverse sample spanning seven countries and two continents provides robust evidence on anaemia prevalence and determinants across varied contexts, enhancing generalizability and enabling cross-regional comparisons rarely available in the literature. The inclusion of both early and late adolescents of both sexes addresses a critical gap, as most previous research has focused narrowly on older adolescent girls. The use of standardized data collection instruments and hemoglobin measurement protocols across sites ensures comparability and minimizes measurement bias.

However, important limitations must be acknowledged. The cross-sectional design precludes causal inference, limiting our ability to determine whether observed associations represent true causal relationships or confounding by unmeasured variables. Dietary assessment relied on food frequency questionnaires without portion size estimation, potentially introducing measurement error and precluding quantitative assessment of nutrient intake adequacy. We lacked data on important potential confounders, including parasitic infections (malaria, helminths), inflammatory conditions, genetic hemoglobinopathies (sickle cell disease, thalassemia), and household-level food security, all of which may independently influence anaemia risk. The study did not assess iron supplementation coverage or adherence, limiting our understanding of program effectiveness. Selection bias may have occurred if adolescents with severe anaemia or those from the most marginalized households were underrepresented in the sample. Finally, while our study provides valuable quantitative evidence, qualitative research is needed to illuminate the social, cultural, and behavioral contexts underlying these quantitative patterns and to inform culturally appropriate intervention design.

## Conclusions

Anaemia affects more than two in five adolescents across Sub-Saharan Africa and India, with particularly alarming prevalence in West African countries. Female sex, younger age, and underweight nutritional status independently predict anaemia, while egg consumption may offer modest protection. As one of the few cross-continental studies examining adolescent anaemia across diverse settings, this research provides an essential preliminary evidence base for designing and scaling effective interventions. Addressing adolescent anaemia requires moving beyond vertical iron supplementation programs toward integrated approaches that combine nutrition-specific interventions with broader investments in health systems, education, and poverty reduction. Only through such comprehensive, sustained efforts can we break the intergenerational cycles of malnutrition and anemia that continue to undermine adolescent health and development in these regions.

## Supporting information

S1 Checklist Inclusivity in global research. (DOCX)
