# Supplementary material for: Prevalence and factors associated with anaemia among adolescents in Sub-Saharan Africa and India
Source: PLOS Glob Public Health. 2026 Jul 31;6(7):e0006963. doi: 10.1371/journal.pgph.0006963 (PMC13426987; doi:10.1371/journal.pgph.0006963)
Supplement: Attachment — Submitted filename: Response to Reviewers.docx [file pgph.0006963.s003.docx]

Department of Population, Family and Reproductive Health,

University of Ghana School of Public Health
P. O. Box LG 13
Legon, Accra
Ghana
[amanu@ug.edu.gh](mailto:amanu@ug.edu.gh)

27 June 2026

The Editor
PLOS Global Public Health

Dear Editor,

**RESPONSE TO REVIEWERS’ COMMENTS**

On behalf of the authors of this manuscript, I would like to thank the editor and the two reviewers for their insightful comments that have significantly shaped the manuscript for public consumption.

I wish to resubmit the revised manuscript titled **“****Prevalence and Factors Associated with Anaemia among Adolescents in Sub-Saharan Africa and India”** for your consideration, having addressed reviewers’ comments point-by-point. For clarity and ease of verification, I have numbered the various comments from the editor and the two reviewers serially in the order they appeared in the email to me as follows:

| **No.** | **Reviewer Comments** | **Response** |
| --- | --- | --- |
| 1. | Please include a complete copy of PLOS’ questionnaire on inclusivity in global research. | An inclusivity questionnaire has been completed and uploaded as Supporting Information (S1 Checklist). In the manuscript, we have inserted a sub-heading “Inclusivity in global research” on page 11, Lines 278-280. |
| 2. | Tables should not be uploaded as individual files. | A table, originally labelled S1 Table, was appended as supporting information to Fig 2, which is a Plot of odds ratios and their 95% confidence intervals from **the multivariable logistic regression model.**  However, based on your recommendation, we have inserted the table in the body of the manuscript labelled Table 3 (see pages 18-19). |
| 3. | Review your reference list to ensure that it is complete and correct. | The reference list has been reviewed thoroughly for completeness and correctness.  Also, five additional references (46-50) have been included as a result of the revision of the discussion session covering haemoglobinopathies and the high prevalence of sickle cell disease in West Africa. |
| 4. | **Abstract:** There is a lack of statistical detail in lines 49–50. Please provide the specific adjusted odds ratios (aORs) and 95% Confidence Intervals (CIs) for the finding: *‘Adolescents with normal or overweight body mass index (BMI) had lower odds of anaemia than their counterparts.’* | Specific adjusted odds ratios (aORs) and their 95% Confidence Intervals (CIs) for the finding have been inserted. The sentence now reads ‘*Adolescents with normal (aOR 0.76; 95%CI: 0.64=0.90) and overweight (aOR 0.79; 95%CI: 0.63-0.97) body mass index (BMI) had lower odds of anaemia than their underweight counterparts.’* (see page 2, Lines 51-52) |
| 5. | While India is prominently featured in the title and introduction, India-specific prevalence data is missing from the results section of the abstract and the results section. To ensure a balanced cross-continental representation, please include the India-specific prevalence (30.9%) alongside the Sub-Saharan African (SSA) results. | India-specific prevalence and cross-continental representative data have been provided in both the results section of the abstract and the results section of the main manuscript (see page 2, Lines 47-48 of the abstract, and page 14, Lines 298-302 of the main manuscript). Details about anaemia prevalence have also been shown in Fig 1 (Panels A, B, and C). |
| 6. | The conclusion states that the "association with normal BMI underscores the need for age-specific strategies." This is slightly confusing as written. If normal/overweight BMI is protective (as stated in lines 49-50), the conclusion should perhaps emphasize that underweight status is the risk factor or clarify if there's a surprising nuance regarding "normal" BMI individuals being at risk? | We very much agree with you. Thus, the conclusions have been modified to reflect the results and for clarity (page 2, Lines 57-61). |
| 7. | **Introduction** - Please address the verbatim repetition in lines 68–69. | The verbatim repetition has been deleted (see Lines 79-80). |
| 8. | Pg5, line 139: The citations are incorrectly cited and are in Harvard format, not Vancouver, as the rest of the article. | The citation has been corrected to Vancouver (Page 5, Line 140). |
| 9. | Although the HemoCue is a validated point-of-care test and easy to use, a paragraph on how the personnel performing this test were trained, including the maintenance and quality control, should be included. Sample collection training and the processing of samples are important. | A paragraph has been inserted in the methods section of the manuscript on how personnel who performed the point-of-care test were trained about the operations of the HemoCue device, how to perform the test, and quality control measures to ensure accuracy and validity (see page 8, Lines 191-200). |
| 10. | **Discussion:** The incidence of haemoglobinopathies and sickle cell anemia, particularly in West African Countries, is only mentioned as part of the limitations, but not discussed. West Africa is the epicenter of sickle cell disease, and this fact could have had a major impact on the results. It could explain the diverse findings, especially in Ghana, Burkina Faso and Nigeria. I would recommend more discussion on this within the body of the discussion when discussing the diverse findings across regions. | Thanks for this observation. This concern has been addressed extensively in the manuscript. A paragraph has been inserted covering a discussion on the issues of haemoglobinopathies and sickle cell anemia disease (see pages 20 and 21, Lines 394-409). |

Please, all correspondence concerning this manuscript must be addressed to [amanu@ug.edu.gh](mailto:amanu@ug.edu.gh).

Thank you, looking forward to hearing from you.

Yours sincerely,

Dr. Adom Manu
