# Supplementary material for: Prevalence and factors associated with anaemia among adolescents in Sub-Saharan Africa and India
Source: PLOS Glob Public Health. 2026 Jul 31;6(7):e0006963. doi: 10.1371/journal.pgph.0006963 (PMC13426987; doi:10.1371/journal.pgph.0006963)
Supplement: Peer review history

# Peer review: Decision Letter 0

**DOI:** 10.1371/journal.pgph.0006963.r001

**Authors:** Takana Silubonde-Moyana

23 Apr 2026

PGPH-D-26-00327

Prevalence and Factors Associated with Anaemia among Adolescents in Sub-Saharan Africa and India

PLOS Global Public Health

Dear Dr. Adom Manu

Thank you for submitting your manuscript to PLOS Global Public Health. After careful consideration, we feel that it has merit but does not fully meet PLOS Global Public Health’s publication criteria as it currently stands. Therefore, we invite you to submit a revised version of the manuscript that addresses the points raised during the review process.

Editor Comments : This is a timely and well-executed cross-continental study that addresses a critical evidence gap in adolescent health. The research provides valuable insights into the "forgotten middle" of public health adolescents aged 10-19, an entry point for nutrition-sensitive policy interventions. The manuscript is written with significant academic gravity and clarity. However, there are a minor technical revisions and clarifications needed.

Please submit your revised manuscript by 7/05/26. If you will need more time than this to complete your revisions, please reply to this message or contact the journal office at globalpubhealth@plos.org. When you're ready to submit your revision, log on to https://www.editorialmanager.com/pgph/ and select the 'Submissions Needing Revision' folder to locate your manuscript file.

Please include the following items when submitting your revised manuscript:

- A letter that responds to each point raised by the editor and reviewer(s). You should upload this letter as a separate file labeled 'Response to Reviewers'.
- A marked-up copy of your manuscript that highlights changes made to the original version. You should upload this as a separate file labeled 'Revised Manuscript with Track Changes'.
- An unmarked version of your revised paper without tracked changes. You should upload this as a separate file labeled 'Manuscript'.

Guidelines for resubmitting your figure files are available below the reviewer comments at the end of this letter.

As the corresponding author, your ORCID iD is verified in the submission system and will appear in the published article. PLOS supports the use of ORCID, and we encourage all coauthors to register for an ORCID iD and use it as well. Please encourage your coauthors to verify their ORCID iD within the submission system before final acceptance, as unverified ORCID iDs will not appear in the published article. *Only*  the individual author can complete the verification step; PLOS staff *cannot*  verify ORCID iDs on behalf of authors.

We look forward to receiving your revised manuscript.

Kind regards,

Takana Mary Silubonde-Moyana, PhD

Guest Editor

PLOS Global Public Health

Journal Requirements:

1. Please include a complete copy of PLOS’ questionnaire on inclusivity in global research in your revised manuscript. Our policy for research in this area aims to improve transparency in the reporting of research performed outside of researchers’ own country or community. The policy applies to researchers who have travelled to a different country to conduct research, research with Indigenous populations or their lands, and research on cultural artefacts. The questionnaire can also be requested at the journal’s discretion for any other submissions, even if these conditions are not met. Please find more information on the policy and a link to download a blank copy of the questionnaire here: https://journals.plos.org/globalpublichealth/s/best-practices-in-research-reporting. Please upload a completed version of your questionnaire as Supporting Information when you resubmit your manuscript.

2. Tables should not be uploaded as individual files. Please remove these files and include the Tables in your manuscript file as editable, cell-based objects. For more information about how to format tables, see our guidelines: [LINK]

https://journals.plos.org/globalpublichealth/s/tables

If the reviewer comments include a recommendation to cite specific previously published works, please review and evaluate these publications to determine whether they are relevant and should be cited. There is no requirement to cite these works unless the editor has indicated otherwise.

Please review your reference list to ensure that it is complete and correct. If you have cited papers that have been retracted, please include the rationale for doing so in the manuscript text, or remove these references and replace them with relevant current references. Any changes to the reference list should be mentioned in the rebuttal letter that accompanies your revised manuscript. If you need to cite a retracted article, indicate the article’s retracted status in the References list and also include a citation and full reference for the retraction notice.

Additional Editor Comments on the manuscript:

**Abstract**

There is a lack of statistical detail in lines 49–50. Please provide the specific adjusted odds ratios (aORs) and 95% Confidence Intervals (CIs) for the finding: *‘Adolescents with normal or overweight body mass index (BMI) had lower odds of anaemia than their counterparts.’*

While India is prominently featured in the title and introduction, India-specific prevalence data is missing from the results section of the abstract and the results section. To ensure a balanced cross-continental representation, please include the India-specific prevalence (30.9%) alongside the Sub-Saharan African (SSA) results.

The conclusion states that the "association with normal BMI underscores the need for age-specific strategies." This is slightly confusing as written. If normal/overweight BMI is protective (as stated in line 49-50), the conclusion should perhaps emphasize that underweight status is the risk factor or clarify if there's a surprising nuance regarding "normal" BMI individuals being at risk?

**Introduction**  _Please address the verbatim repetition in lines 68–69: *“...where systemic health inequities, nutritional deficiencies, and infectious diseases compound the problem [1], where systemic health inequities, nutritional deficiencies, and infectious diseases compound the problem.”*  The second instance of this phrase should be deleted.

[Note: HTML markup is below. Please do not edit.]

Reviewers' comments:

Reviewer's Responses to Questions

**Comments to the Author**

1. Does this manuscript meet PLOS Global Public Health’s publication criteria? Is the manuscript technically sound, and do the data support the conclusions? The manuscript must describe methodologically and ethically rigorous research with conclusions that are appropriately drawn based on the data presented.

Reviewer #1: Yes

**********

2. Has the statistical analysis been performed appropriately and rigorously?

Reviewer #1: Yes

**********

3. Have the authors made all data underlying the findings in their manuscript fully available (please refer to the Data Availability Statement at the start of the manuscript PDF file)?

The PLOS Data policy requires authors to make all data underlying the findings described in their manuscript fully available without restriction, with rare exception. The data should be provided as part of the manuscript or its supporting information, or deposited to a public repository. For example, in addition to summary statistics, the data points behind means, medians and variance measures should be available. If there are restrictions on publicly sharing data—e.g. participant privacy or use of data from a third party—those must be specified.

Reviewer #1: Yes

**********

4. Is the manuscript presented in an intelligible fashion and written in standard English?

PLOS Global Public Health does not copyedit accepted manuscripts, so the language in submitted articles must be clear, correct, and unambiguous. Any typographical or grammatical errors should be corrected at revision, so please note any specific errors here.

Reviewer #1: Yes

**********

5. Review Comments to the Author

Please use the space provided to explain your answers to the questions above. You may also include additional comments for the author, including concerns about dual publication, research ethics, or publication ethics. (Please upload your review as an attachment if it exceeds 20,000 characters)

Reviewer #1: This article correctly highlights the fact that globally, anemia remains a concern particularly in low to middle income countries. Although this has been studied extensively, there is a lack of data on adolescents, and it is this gap that this study has aimed to address. The article focusses mainly on nutritional anemias, correlating the results with nutritional requirements and other demographics. The strengths of the study are that a large number of participants have been included, and it has studied the prevalence across continents including India and several countries in Africa. This work is relevant and certainly adds to the knowledge on the prevalence of global anemia patterns.

The introduction is well written and certainly motivates the reason for the study and also its relevance. One small comment is that it is very long, but this is a minor observation.

Two other minor corrections are the following:

1. Pg2, line69: There is repetition of the last phrase which starts: " Where systemic health.....

2. Pg5, line139: The citations are incorrectly cited and are in Harvard format not Vancouver as the rest of the article.

The method section is well described, easy to follow and the methods used to gather the data are appropriate for the study. The authors have used standardized questionnaires and have utilized the HemoCue to determine hemoglobin levels. The statistics and data analysis section is well written and appropriate. The only correction for this section is highlighted below.

1. Although the HemoCue is a validated point of care test and easy to use, a paragraph on how the personnel performing this test were trained, including the maintenance and quality control should be included. Sample collection training and the processing of samples is important. As this is the core method and only method used to determine the hemoglobin levels, a more detailed description is required.

Ethical approval has been given from all countries and consent has been obtained from all participants, including guardians if underage. There are not ethical concerns.

The result section is well written and easy to follow. There are no recommended changes to this section.

The discussion is also well structured and comparisons with other studies as well as the final recommendations and conclusions are clear. One recommendation is highlighted below.

1. The incidence of haemoglobinopathies and sickle cell anemia, particularly in West African Countries, is only mentioned as part of the limitations, but not discussed. West Africa is the epicenter of sickle cell disease, and this fact could have had a major impact on the results. It could explain the diverse findings especially in Ghana, Burkina Faso and Nigeria. I would recommend more discussion on this within the body of the discussion, when discussing the diverse findings across regions.

Overall, despite these recommendations and corrections, this article makes a significant contribution to our knowledge on anemia, especially among adolescents. The conclusions are relevant and tie in with the result and method sections.

**********

6. PLOS authors have the option to publish the peer review history of their article (what does this mean?). If published, this will include your full peer review and any attached files.

**Do you want your identity to be public for this peer review?**  If you choose “no”, your identity will remain anonymous but your review may still be made public.

For information about this choice, including consent withdrawal, please see our Privacy Policy.

Reviewer #1: **Yes:**  Glenda M. Davison

**********

[NOTE: If reviewer comments were submitted as an attachment file, they will be attached to this email and accessible via the submission site. Please log into your account, locate the manuscript record, and check for the action link "View Attachments". If this link does not appear, there are no attachment files.]

Figure Resubmissions:

While revising your submission, we strongly recommend that you use PLOS’s NAAS tool (https://ngplosjournals.pagemajik.ai/artanalysis) to test your figure files. NAAS can convert your figure files to the TIFF file type and meet basic requirements (such as print size, resolution), or provide you with a report on issues that do not meet our requirements and that NAAS cannot fix.

After uploading your figures to PLOS’s NAAS tool - https://ngplosjournals.pagemajik.ai/artanalysis, NAAS will process the files provided and display the results in the "Uploaded Files" section of the page as the processing is complete. If the uploaded figures meet our requirements (or NAAS is able to fix the files to meet our requirements), the figure will be marked as "fixed" above. If NAAS is unable to fix the files, a red "failed" label will appear above. When NAAS has confirmed that the figure files meet our requirements, please download the file via the download option, and include these NAAS processed figure files when submitting your revised manuscript.

# Author comment: Author response to Decision Letter 1

**DOI:** 10.1371/journal.pgph.0006963.r002

3 Jul 2026

Attachment Submitted filename: Response to Reviewers.docx

# Editor report: Decision Letter 1

**DOI:** 10.1371/journal.pgph.0006963.r003

**Authors:** Takana Silubonde-Moyana

13 Jul 2026

Prevalence and Factors Associated with Anaemia among Adolescents in Sub-Saharan Africa and India

PGPH-D-26-00327R1

Dear Dr Adom Manu

We are pleased to inform you that your manuscript 'Prevalence and Factors Associated with Anaemia among Adolescents in Sub-Saharan Africa and India' has been provisionally accepted for publication in PLOS Global Public Health.

Before your manuscript can be formally accepted you will need to complete some formatting changes, which you will receive in a follow up email. A member of our team will be in touch with a set of requests.

Please note that your manuscript will not be scheduled for publication until you have made the required changes, so a swift response is appreciated.

IMPORTANT: The editorial review process is now complete. PLOS will only permit corrections to spelling, formatting or significant scientific errors from this point onwards. Requests for major changes, or any which affect the scientific understanding of your work, will cause delays to the publication date of your manuscript.

If your institution or institutions have a press office, please notify them about your upcoming paper to help maximize its impact. If they'll be preparing press materials, please inform our press team as soon as possible -- no later than 48 hours after receiving the formal acceptance. Your manuscript will remain under strict press embargo until 2 pm Eastern Time on the date of publication. For more information, please contact globalpubhealth@plos.org.

Thank you again for supporting Open Access publishing; we are looking forward to publishing your work in PLOS Global Public Health.

Best regards,

Takana Mary Silubonde-Moyana, PhD

Guest Editor

PLOS Global Public Health

********************************************************
